# Remote Ischemic Conditioning in Cerebral Diseases and Neurointerventional Procedures: Recent Research Progress

**DOI:** 10.3389/fneur.2018.00339

**Published:** 2018-05-16

**Authors:** Geng Zhou, Ming Hua Li, Gabriel Tudor, Hai Tao Lu, Ramanathan Kadirvel, David Kallmes

**Affiliations:** ^1^Shanghai Jiao Tong University Affiliated Sixth People’s Hospital, Shanghai, China; ^2^Mayo Clinic, Rochester, MN, United States

**Keywords:** remote ischemic conditioning, acute ischemic stroke, ischemia/reperfusion injury, neuroprotection, neurointerventional procedures

## Abstract

Cerebral ischemia and stroke are increasing in prevalence and are among the leading causes of morbidity and mortality in both developed and developing countries. Despite the progress in endovascular treatment, ischemia/reperfusion (IR) injury is an important contributor to post-surgical mortality and morbidity affecting a wide range of neurointerventional procedures. However, pharmacological recruitment of effective cerebral protective signaling has been largely disappointing to date. In remote ischemic conditioning (RIC), repetitive transient mechanical obstruction of vessels at a limb remote from the IR injury site protects vital organs from IR injury and confers infarction size reduction following prolonged arterial occlusion. Results of pharmacologic agents appear to be species specific, while RIC is based on the neuroprotective influences of phosphorylated protein kinase B, signaling proteins, nitric oxide, and transcriptional activators, the benefits of which have been confirmed in many species. Inducing RIC protection in patients undergoing cerebral vascular surgery or those who are at high risk of brain injury has been the subject of research and has been enacted in clinical settings. Its simplicity and non-invasive nature, as well as the flexibility of the timing of RIC stimulus, also makes it feasible to apply alongside neurointerventional procedures. Furthermore, despite nonuniform RIC protocols, emerging literature demonstrates improved clinical outcomes. The aims of this article are to summarize the potential mechanisms underlying different forms of conditioning, to explore the current translation of this paradigm from laboratory to neurovascular diseases, and to outline applications for patient care.

## Introduction

Recent studies show that ischemia/reperfusion (IR) injury is an important contributor to post-surgical mortality and morbidity affecting those undergoing a wide range of neurointerventional procedures (1, 2). Effective protection attenuating IR injury is therefore an important factor in improving patient prognosis. However, pharmacological strategy to protect the brain against IR injury has been largely disappointing to date.

Ischemic conditioning, a powerful non-pharmacological strategy for reducing IR injury, was recognized in animal models in 1986 (3), though this innate cytoprotective mechanism in the brain was noted as early as the 1940s (4). By 1996, its use extended to organs remote from the heart in the form of remote ischemic conditioning (RIC) (5). Today, RIC is a remarkably simple and low-cost intervention that employs repetitive inflation and deflation of a standard arm or leg blood pressure cuff and constitutes a highly effective therapy for protecting vital organs from IR injury. Base on its simplicity, accessibility, and non-invasive nature, RIC has the potential for treatment in a wide variety of conditions including acute, subacute, and chronic neurological diseases with an ischemic basis, such as acute ischemic stroke (AIS) (6).

The aims of this article are to summarize the potential mechanisms underlying different forms of conditioning, to explore the current translation of this paradigm from laboratory to neurovascular diseases, and to outline applications for patient care.

## RIC Protocol

The most effective RIC protocol has yet to be fully defined. Currently, the most commonly employed technique across clinical settings is three to four repetitions of 5-min inflation/deflation using a standard blood pressure cuff. Tourniquet pressure should be above the systolic pressure to ensure arterial occlusion. Its localization (arm versus thigh) does not affect cytoprotection (7). However, more than eight ischemic cycles or cycles >10 min did not lead to better results and possibly even increased injury in mice (8). If RIC were considered in the manner one would analyze a therapeutic drug, its exact dosage, pharmacokinetics, and pharmacodynamics would remain largely unclear.

**Figure 1:**
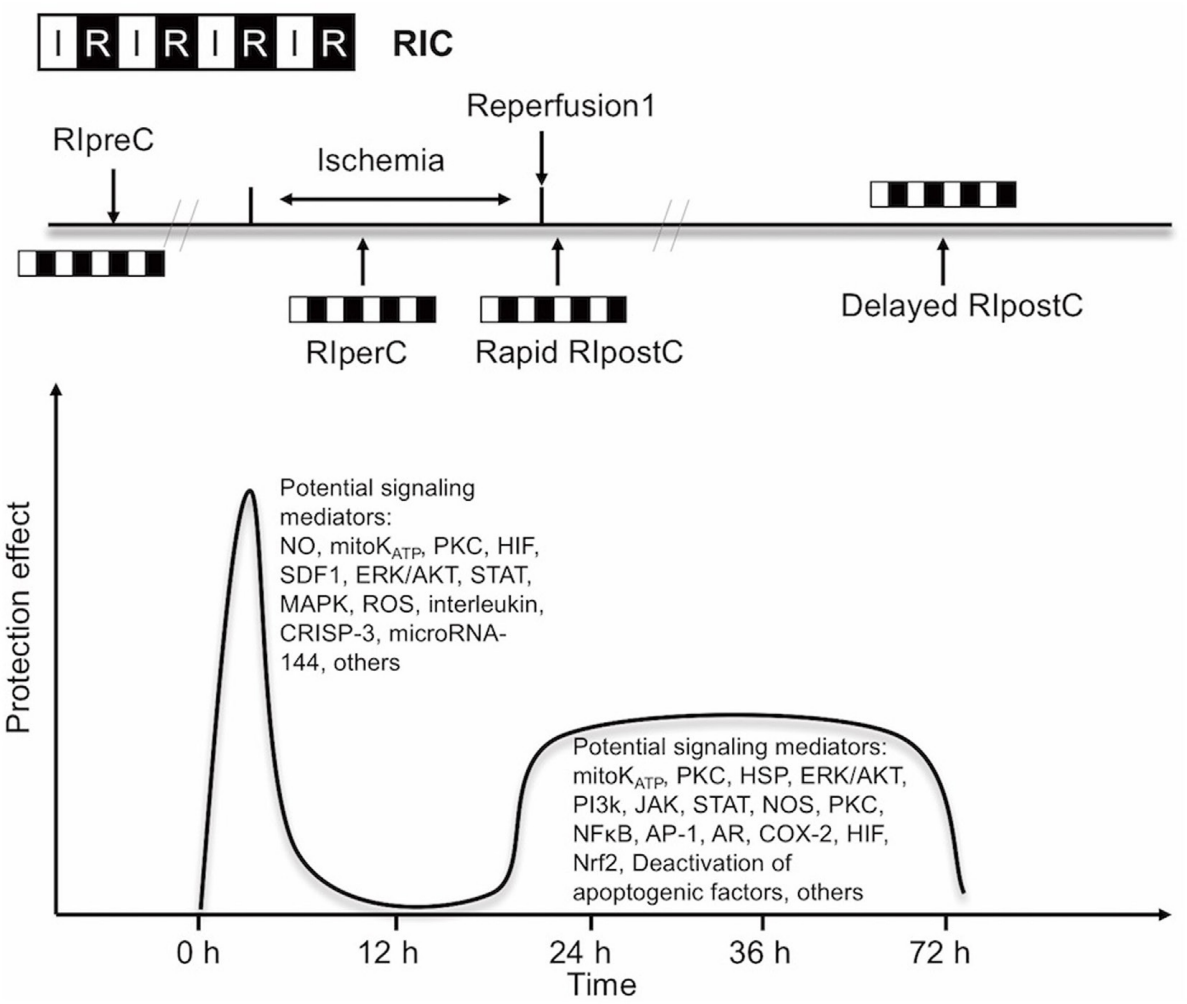
Simplified scheme and possible mechanisms of the temporal nature of the two windows of remote ischemic conditioning (RIC). Abbreviations: AR, aldose reductase; AP-1, activator protein 1; COX-2, cyclooxygenase-2; CRISP-3, cysteine-rich secretory protein 3; NOS, nitric oxide synthase; ERK/AKT, extracellular signal regulated kinase/protein kinase B; HIF, hypoxia-inducible factor; HSP, heat shock protein; JAK, Janus kinase; K_ATP_, ATP-sensitive potassium channel; MAPK, mitogen-activated protein kinase; Mito, mitochondria; NFκB, nuclear factor κB; NO, nitric oxide; Nrf2, nuclear factor erythroid 2-related factor; PI3k, phosphoinositide-3 kinase; PKC, protein kinase C; ROS, reactive oxygen species; SDF1, stromal cell-derived factor 1; STAT, signal transducer and activator of transcription.

Experimental and clinical evidence suggests that RIC, as well as other preconditioning stimuli, activates at least two distinct time frames of protection against IR injury of brain and heart. The time window of brain protection by preconditioning has also been demonstrated *in vitro* model (9). The initial time window of brain protection is short lasting as a result of changes in ion channel permeabilities, protein phosphorylation, and release of several mediators [including adenosine and bradykinin (BK)]. It occurs immediately after the RIC stimulus and lasts 2 h (10). The delayed form of protection, referred to as the second window of protection (SWOP), follows 12–24 h later, and lasts 48–72 h (as shown across multiple species) (11). SWOP may be triggered by the reactive oxygen species (ROS) and mediated by modulated inflammatory response, improved endothelial function, and activation of gene expression (such as HIF, toll-like receptor caspases, and heat shock proteins) (Figure 1) (12, 13). Various clinical studies have demonstrated the SWOP in RIC, although all the studies are in cardiac surgery settings (14).

The concept of RIC has now expanded into three temporal variants after its initial application: remote ischemic preconditioning (RIPreC), perconditioning (RIPerC), and postconditioning (RIPostC) (15–17). Brain mechanisms are independent of the timing of conditioning strategies (pre-, per-, postconditioning), and their effects have a great deal of overlap.

## RIC Mechanisms

**Figure 2:**
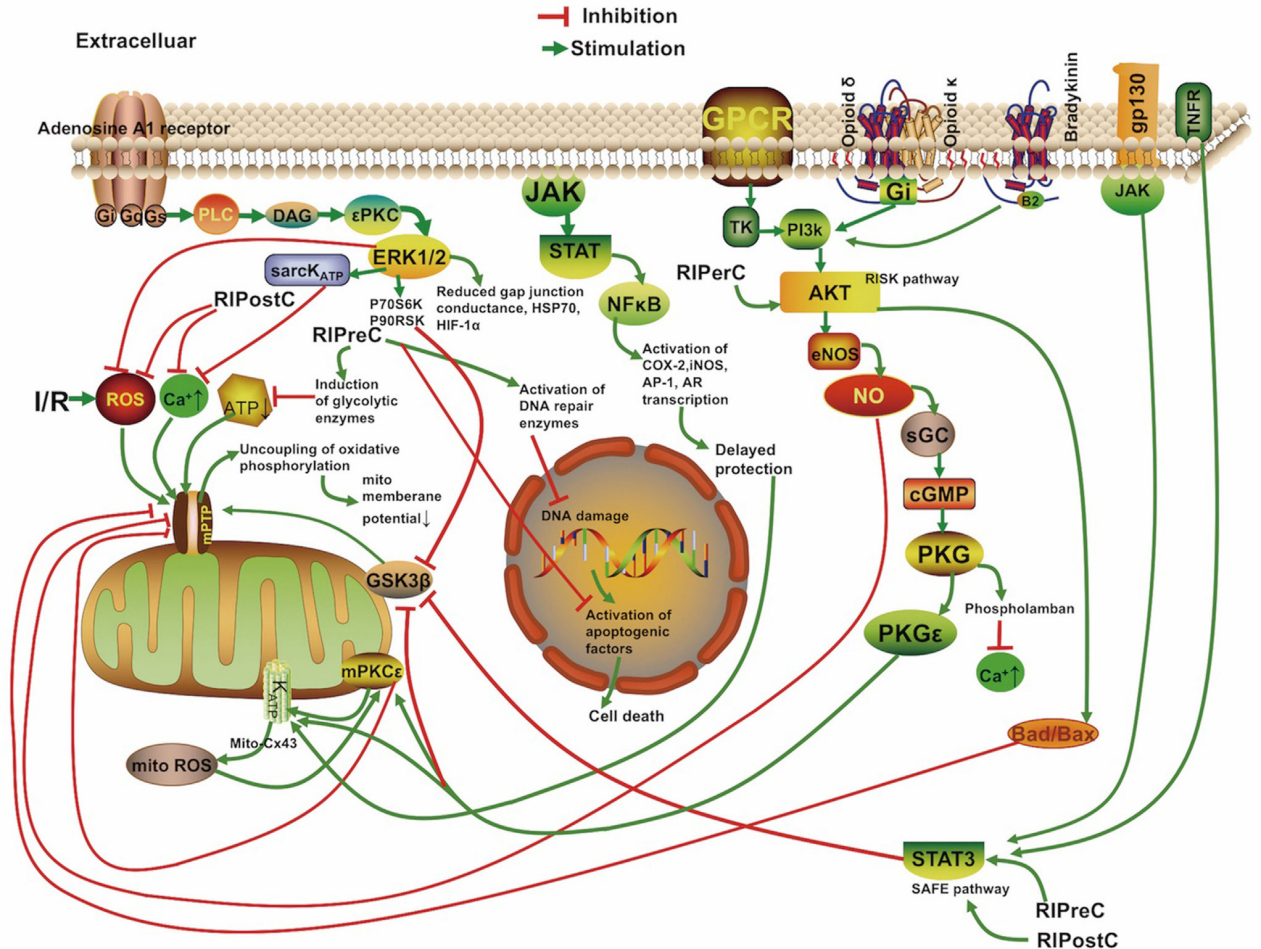
Overview of the proposed signaling cascades recruited in the setting of remote ischemic conditioning based on available data. Abbreviations: Akt, protein kinase B; AR, aldose reductase; AP-1, activator protein 1; cGMP, cyclic guanosine monophosphate; COX-2, cyclooxygenase-2; Cx 43, connexin 43; DAG, diacylglycerol; eNOS, endothelial nitric oxide synthase; ERK, extracellular signal regulated kinase; Gs/Gi/q, stimulatory/inhibitory G protein; GPCR, G protein-coupled receptor; gp130, glycoprotein 130; GSK3β, glycogen synthase kinase 3 β; HIF-1α, hypoxia-inducible factor 1α; HSP, heat shock protein; IR, ischemia/reperfusion; iNOS, inducible nitric oxide synthase; JAK, Janus kinase; K_ATP_, ATP-sensitive potassium channel; mPTP, mitochondrial permeability transition pore; Mito, mitochondria; NFκB, nuclear factor κB; NO, nitric oxide; P70S6K, p70 ribosomal S6 protein kinase; P90RSK, 90 ribosomal S6 kinase; PI3k, phosphoinositide-3 kinase; PKC, protein kinase C; PKG, protein kinase G; PLC, phospholipase C; RISK, reperfusion injury salvage kinase pathway; ROS, reactive oxygen species; sarcK_ATP_, sarcolemmal potassium channels; sGC, soluble guanylate cyclase; SAFE, survivor activating factor enhancement; STAT, signal transducer and activator of transcription; TK, tyrosine kinase; TNFR, tumor necrosis factor receptor.

The mechanisms underlying RIC include neurovascular protection, anti-inflammatory action, reduced excitotoxicity, and metabolic protection, which are associated with influences on mitochondria, circulating inflammatory cells, or transcriptional upregulation of protective pathways (Figure 2) (18, 19). There is a consensus that the infarct-sparing effect of all forms of ischemic conditioning involves the upregulation of several signal transduction cascades, which serve to stabilize the mitochondria (20).

Although neurons are assumed to be the cellular target of cerebral conditioning, ischemic tolerance occurring at the level of endothelial and smooth muscle cells contributes to neuronal protection (21). RIPreC was first shown to protect against endothelial injury during IR in humans in 2002 (22), and vasodilation was shown to be better preserved in a preconditioned brain (23). Trans-cranial Doppler measurements of patients undergoing RIC indicated transient cerebral vasodilation over the duration of conditioning (24). All temporal variants of RIC have been proven to prolong protein kinase B (Akt) activity in the endothelium, which increases nitric oxide (NO) production through improved endothelial nitric oxide synthase (eNOS) activity and helps to maintain vascular homeostasis (25–27).

### Cell-Level Mechanisms Underlying RIPreC

The mechanism of brain preconditioning involves a shift in the neuronal excitotoxic/inhibitory balance and a reduction in inflammatory sequelae. Several intracellular signaling pathways and various intercellular mediators and kinases have been identified in tissue protection by RIC. The protective reperfusion injury salvage kinase pathway (RISK) including the phosphoinositide-3 kinase/Akt signaling cascade and the pro-survival survivor activating factor enhancement (SAFE) pathway including the Janus kinase 2 (JAK2)/signal transducer and activator of transcription (STAT)3 signaling cascade are the most important pathways involved in ischemia cytoprotection and eNOS activation (28, 29). And the SAFE pathway was shown to lead to tissue protection independently of the RISK pathway (28). Phosphorylation of JAK2, STAT3, STAT5, Akt, and other signaling complexes may ultimately reduce apoptosis, ROS production, and inflammation (30, 31). In addition, STAT3 located in the matrix of subsarcolemmal and interfibrillar mitochondria also serves to improve mitochondrial respiration and attenuate mPTP opening, and ROS formation (32, 33). And Akt activation, in interaction with STAT3 activation, was mandatory for ischemic preconditioning (34). The activation of the STATs also results in transcriptional upregulation of inducible nitric oxide synthase (iNOS) and cyclooxygenase-2, known distal mediators/effectors of protection (35, 36). There are direct evidences for STATs involvement in patients with RIC (37, 38). A recent study demonstrated that RIPreC could enhance the phosphorylated Akt, STAT3, STAT5, and eNOS expression levels and activating the pro-survival signaling pathway in humans (39). In addition, previous reports showed that NO, hypoxia-inducible factor (HIFs), erythropoietin, free radicals, BK, adenosine, opioids, activation of the ATP-sensitive potassium (K_ATP_) channel, and norepinephrine all have roles in RIPreC (40–42). One of the key regulators of the genomic response after RIPreC is the transcriptional activator HIF. HIF-1 activation is neuroprotective, and a neuron-specific HIF-1α deletion demonstrated exacerbation of brain injury in an experimental model of stroke (43). The growth of new vessels stimulated by the VEGF and erythropoietin cytokines are also regulated by HIF-1 (43). Some researchers believe that expression of HIF-1α—but not phosphorylation of extracellular signal regulated kinase 1/2 (ERK1/2), Akt, or STAT5—is required for RIPreC (44). Inflammatory mediators, such as interleukin-6, tumor necrosis factor (TNF), intracellular adhesion molecule, matrix metalloproteinase 9, and C-reactive protein are downregulated through RIPreC (45).

Microarrays indicate that preconditioning stimulates a genomic reprogramming of cells that confers cytoprotection, recovery, neurogenesis, and angiogenesis (46). In particular, genes regulating cell metabolism, signal transport, growth factors, ion channels, metallothionins, or cell cycle/apoptosis are selectively upregulated (46, 47). The microRNA for glutamate receptor, ionotropic delta 2, was reported to be downregulated in the mouse brain after RIPreC (46).

Using a global model of ischemia preconditioning in gerbils, short stimuli were shown to induce an increase in dendritic spine density of vulnerable hippocampal CA1 neurons 3 days after reperfusion, comparable to the SWOP of the neuroprotective effect induced by preconditioning (48). Preconditioning in immature brains also increases the concentration of astrocytic glycogen, which is neuroprotective, and delays energy depletion caused by ischemia (49). Moncada found that preconditioning increases expression of cyclooxygenase 1 and prostacyclin synthase; these enzymes act successively to produce prostacyclin, which inhibits platelet aggregation and vasoconstriction (50). Røpcke et al. also demonstrated that RIPreC reduces arterial thrombus formation and embolization in rats (51). Several clinical trials are underway to test the safety and efficacy of RIPreC for protecting the brain against anticipated damage (52, 53), and its procedural simplicity makes it an excellent candidate for study in future clinical trials.

#### Corroborating Evidence Based on Transient Ischemic Attack (TIA) Neuroprotection

Patients who suffer a TIA show better clinical outcomes in subsequent strokes compared to those who suffer similar strokes without first having suffered a TIA, which may be due to activation of the same neuroprotective pathways as RIPreC (54). Schaller found that stroke patients showed more favorable neurological outcomes when the preceding TIAs occurred 1–7 days prior to stroke (55). Similarly, in a German study comprised of 7,611 patients, TIA was associated with reduced stroke severity (56). Recent data also suggests that peripheral vascular disease with chronic limb hypoperfusion was associated with less disability and lower mortality in AIS (57). In contrast to the findings, Kim et al. reported that a low ankle-brachial blood pressure index (ABI) (<0.9) was associated with an increased risk of poor functional outcome in patients with acute cerebral infarction (odds ratio 3.452, *P* < 0.001) than patients without low ABI (58). However, in this study, the patients with a low ABI were more likely to have a high NIHSS score at baseline. Besides, the patients with a low ABI more often had diabetes mellitus (44.9 versus 29.5%, *P* = 0.007). Diabetes mellitus itself may attenuate the effectiveness of RIC (59). In future trials, subgroup analysis of patients with comorbidities such as diabetes is needed.

### Alternative Method: RIPerC

Remote ischemic preconditioning may be not practical in acute clinical settings because it must be initiated before the ischemic event. The neuroprotective efficacy of RIPerC has been proven in a number of animal models (10, 14, 25, 60). Furthermore, mild to moderate hemorrhage after tissue plasminogen activator (tPA) was attenuated when RIPerC therapy was performed 2 h before tPA infusion, making it an excellent candidate for combination therapy with tPA (61). Clinical MRI evidence suggests RIPerC treatment induces an immediate neuroprotective effect by reducing cytotoxic cerebral edema when perfusion is restored (62). RIPerC also upregulates mRNA expression of eNOS about 10-fold in the blood vessels, from the site of conditioning, and increases the concentration of NO in plasma (63).

### The Reasoning Behind RIPostC

Remote ischemic postconditioning can be used in both elective and acute settings. Evidence from experimental and trial studies supports an additive protective effect of combined RIPreC and postconditioning, as reperfusion itself is associated with cell injury and cell death in its very early moments (64–66). Postconditioning likely mitigates damage from sudden reperfusion, plausibly blocking production of ROS and reactive nitrogen species and thus attenuating reperfusion-induced brain injury (67), or possibly by attenuating endoplasmic reticulum stress response-induced apoptosis (68). The pro-survival protein kinases extracellular signal-regulated kinases (ERK), p38 mitogen-activated protein kinase (MAPK), and Akt showed prolonged phosphorylation in the cortex of postconditioned rats (69). Protection from RIPostC is blocked in animal models by removing the influence of STAT3 and mitochondrial K_ATP_ channels, as well as TNF α (33, 70).

## Mitochondria and RIC

Mitochondria play critical roles in all pathways triggered by RIC. RIC causes recruitment of ligands such as adenosine and opioids to Gprotein-coupled receptors. This action leads to the activation of signaling protein kinases and the opening of mitochondrial K_ATP_ channels, which subsequently prevents the opening of the mitochondrial permeability transition pore (mPTP) after the first minutes of reperfusion whereby tissue protection is activated (71–73).

The role of signal transduction pathways during RIC has predominately been demonstrated in the heart. However, the presence of STATs in the mitochondria was confirmed in a number of organs including heart, kidney, and brain (74). A few reports in the literature have suggested the involvement of MAPKs, Akt, HIF-1α, and STATs in mitochondrial neuroprotection following preconditioning (30, 75–77). STATs have been shown to regulate mitochondrial function by preserving efficiency of electron transport chain complexes (35, 78).

## Transfer of the Cerebral Protective Stimulus

In RIC, transient, reversible episodes of ischemia with reperfusion in the stimulus location render remote tissues and target organs resistant to IR injury. At present, transfer of the cerebral protective stimulus is not well understood, though studies have shown it to act through multiple pathways (15).

### Humoral Pathways

The humoral pathway has been most extensively studied. Some studies have identified specific factors, such as stromal cell-derived factor-1 α, interleukin, nitrite, cysteine-rich secretory protein 3, and microRNA-144 as possible candidate transfer factors (51, 79, 80). Ueno et al. suggest that RIPreC transiently increases plasma VEGF levels by downregulating miR-762 and miR-3072-5p in CD34-positive bone marrow cells, leading to protection against organ ischemia (81). In a recent human study, only STAT5 signaling was identified to be associated with RIPreC humoral transfer (38). Endothelial cells were suggested as the target for RIPreC-released mediators (82). Finally, Dong et al. suggest that humoral factors, rather than the neural pathway, play an important role in the formation of the tolerance against spinal cord ischemia by limb RIPreC (83).

### Nerve Pathway

Occlusion with a tourniquet on the arm can stimulate the release of autacoids that activate an afferent neural pathway and/or cause the release of NO from blood vessels (80, 84, 85). Transection of the femoral nerve or spinal cord can abrogate the effect of RIC in rabbits (86). The dependence of remote conditioning on intact neural pathways also may explain why its effects seem to be attenuated in patients with neuropathy (87).

Mastitskaya et al.’s study used viral gene transfer and optogenetics to show that the dorsal motor neurons of the vagus in the brainstem were required for RIPreC to have a cardioprotective effect, and that stimulation of these neurons mimicked the effect of RIPreC (88). Interestingly, femoral nerve or sciatic nerve resection alone only partially abolished the infarct-limiting effect of RIPreC in mice, suggesting the influence of both neural and humoral pathways (89).

### Inflammatory Pathway

Remote ischemic preconditioning has been shown to have a systemic anti-inflammatory influence through upregulation of cytoprotective genes and suppression of proinflammatory genes in immune cells (90). Circulating monocytes and neutrophil infiltration play a key role in IR injury. RIPreC downregulated the expression of a broad spectrum of proinflammatory genes in circulating monocytes. For circulating neutrophil, RIPreC activated signal pathways in neutrophils modulating the release of proinflammatory cytokines and the expression of adhesion markers. Consequently, RIPreC negatively affected their function (18). Microarray analysis showed that reduction of inflammatory gene expression takes place within 15 min of RIC and at 24 h after conditioning in humans (18). Humoral, neural, and anti-inflammatory pathways probably interact with each other and are not necessarily mutually exclusive (91).

## Clinical Applications

Larger trials of RIC, especially for cardioprotection but also for kidney and neuroprotection, have largely supported the consensus of RIC’s lack of harmful influence and reduction of IR injury when established protocols are used and in the absence of propofol (6, 92). Several clinical studies are also underway to expand the literature on neuroprotection specifically (52, 53).

### RIC in AIS

Over 10 million people worldwide suffer an AIS each year (93), yet few neuroprotective treatments against IR injury have been proven effective: clinical trials of more than 50 compounds for treatment of IR injury secondary to AIS all showed negative results. Mechanical thrombectomy has been widely accepted as an effective treatment for AIS. Despite the sharp increase in recanalization rate with current thrombectomy devices compared with tPA, cerebral reperfusion after endovascular embolectomy and/or tPA may cause deterioration of penumbra, disruption of the blood–brain barrier, cerebral edema, and intracerebral hemorrhage (94). Thus, there is an urgent need for effective forms of secondary prevention after the acute phase of AIS intervention, for which RIC is an excellent candidate.

In a model of autologous thromboembolic clots, RIPerC has been effective in mice models when applied 2 h after stroke onset with or without late (4 h after stroke onset) intravenous (IV) tPA (25). Hahn et al. show that infarct size in a rat AIS model was reduced by RIPreC but even further by RIPerC (17). In an analogous study, RIPerC therapy also improved the cerebral blood flow (CBF) and the hemorrhage, edema, and neurobehavioral outcomes significantly on top of the reduction in infarction size compared to IV-tPA alone at 4 h post-stroke (95). Hess et al. show optimal results occurred when RIPerC was started as soon as possible after stroke onset and RIPostC was administered two to three times during first day and repeated daily during the following week (96).

#### Trials in AIS

Several trials studying the effect of RIC on AIS patient outcomes have shown benefits when RIC is administered during ischemia. Hougaard et al. (62) found an overall reduction in the risk of infarction for tissue subjected to pre-hospital RIPerC at 1 month but the study was not powered to show effect in clinical outcome at 3 months. The Remote Ischemic Conditioning After Stroke Trial study (64), a blinded placebo-controlled trial of RIC in AIS patients, showed improved clinical outcome in the RIC group. Compared with sham, 90-day NIHSS score was significantly lower in the RIC group (1 versus 3, *P* = 0.04). RIC also increased plasma heat shock protein 27 (HSP27, *P* < 0.05) level in the study, compared with control. The investigators suggested that the neuroprotective effects may be mediated through phosphorylated HSP27. A research group in Denmark administered RIPerC during transportation in the ambulance as a pretreatment to IV alteplase. Overall, the study showed RIPerC to be safe and feasible in the setting of AIS, with the likely benefit of greater tissue survival in the penumbra than the control (62). Another randomized trial also found that high prestroke physical activity is associated with reduced infarct size after IV tPA treatment only in patients receiving adjuvant RIPerC (97). While a French multicentric trial of RIC for ischemic stroke within 6 h of symptom onset is currently underway. Results of this trial have not yet been reported (98).

### Other Clinical Applications for RIC

#### Intracranial Atherosclerotic Stenosis

Endovascular treatment of ICAS carries a risk of intraoperative and postoperative ischemic events, allowing for non-urgent consideration of protection against IR injury. RIPreC alone was recently found to significantly decrease the incidence of stroke in patients with ICAS (26.7 versus 7.9%), increase CBF, and protect against ischemia-related neurological morbidity (99). Meng et al. (99) found that RIC could improve the cerebral circulation in patients with intracranial arterial stenosis. While RIC was also reported to be effective in cerebral small vessel disease (SVD) related cognitive impairment. Wang et al. (100) randomly assigned 30 patients with mild cognitive impairment caused by cerebral SVD to receive RIC (by the method used by Meng et al. twice daily for 12 months) or to receive a sham intervention; the patients who received RIC had a higher reduction of white matter hyperintensities volume (−2.632 versus −0.935 cm^3^, *P* = 0.049), with a better visuospatial and executive ability at 1 year (0.639 versus 0.191, *P* = 0.048). Meanwhile, in a bilateral carotid artery stenosis mouse model with vascular cognitive impairment, RIC was effective in improving cognition and CBF, attenuating tissue damage (101).

#### Carotid Artery Stenting (CAS)

Carotid artery stenting is a selective procedure used to tread carotid artery stenosis, RIC has been evaluated in surgical brain injury paradigms such as hypothermic circulatory arrest and following carotid endarterectomy. Though a pilot study of 70 patients who received RIC showed no statistically significant improvement in neurological outcome (53), the first proof-of-concept trial of RIC before CAS found that RIC can ameliorate the complications of distal thromboembolization (102). This is the first study to show effect of RIC given before CAS on ischemic lesions size and number assessed by MRI. The authors reported that the incidence of new ischemic lesions were lower in patients who received RIC than in patients who did not (15.87 versus 36.51%, *P* < 0.01), with smaller infarct volume (0.06 versus 0.17 ml).

#### Subarachnoid Hemorrhage (SAH) From Intracranial Aneurysm

The leading cause of SAH is rupture of an intracranial aneurysm, accounting for roughly 80% of cases. Even if embolization of the ruptured intracranial aneurysm is successful, delayed cerebral ischemia may occur (103). Preconditioning before the induction of SAH in rats was shown to improve vasospasm, reduce cerebral inflammatory cytokines, attenuate tissue hypoxia, and prevent neurological deterioration (51). Some authors believe that SAH is a particularly feasible clinical setting to evaluate human response because RIPreC activates multiple pathways that have been invoked in SAH (104).

Laiwalla et al. reported a matched cohort analysis of RIPostC for patients with aSAH.

Remote ischemic conditioning was independently associated with good outcomes and lower incidence of delayed cerebral ischemia (105). A longitudinal human pilot study in aSAH patients undergoing RIC found coordinated expression and methylation of a small set of key genes in mitotic cell cycle, defense, and inflammatory responses after RIC (106). Other human studies have confirmed the safety and feasibility of lower limb RIC in individuals with aSAH in which no patient experienced delayed cerebral ischemia (51).

## Limitations of RIC

Remote ischemic conditioning can be initiated during pre-hospital transport, through which the patient would receive benefit during triage, imaging, and reperfusion therapy by IV or endovascular methods with low known risk of adverse effects. In the study by Botker et al. (107), the RIC stimulus was initiated in ambulance during transfer for angioplasty, resulting in increased myocardial salvage (36%). RIC intervention can also be delivered on immediate arrival at interventional center when ambulance transit times are short, and even at the onset of reperfusion (108). However, most of the current trials are studies mainly focusing on cardioprotective effects. These studies provided further opportunities to investigate the neuroprotective effect of limb RIC applied in an ambulance, helicopter, or emergency departments, in advance of interventional reperfusion. Moreover, preclinical trial in murine thromboembolic stroke model and pilot trials suggest that RIC can be combined with recombinant tissue plasminogen activator in the pre-hospital setting to increase the protective effect. In the Denmark trial, patients were randomly assigned to receive or not receive RIPerC treatment, and RIPerC was completed during transportation in the ambulance before a final diagnosis of ischemic stroke (62, 109). However, it has been reported that about 3% patients will not able to tolerate tourniquet inflation on their arm (94). Furthermore, RIC would also predetermine the arm to be used for arterial and venous access. Other considerations include the influence on obtaining endovascular access during vascular intervention (110). Finally, the time window and the primary RIC protocol in neuroprotection are still not fully determined.

In two large trials, the benefits from RIC were not confirmed in patients undergoing cardiovascular surgery. However, a point of critique in their studies is that the use of propofol anesthesia in most (111) or all patients (112). The second problem is the inclusion of many patients who also underwent valve surgery. RIC protects only from IR injury and not from traumatic injury at the target organ. Propofol is known to disrupt RIC (113–115). Neither RIC cardioprotection nor STAT5 activation were observed under propofol anesthesia (115). In clinical studies reporting protective effects of RIC, the RIC procedure was either completed without anesthetic intervention or completed during anesthesia induction with anesthetics other than propofol (116). The use of propofol has been suggested to be avoided in future studies on RIC (117). And the efficacy of RIC could also be influenced by many other variables including conditioning protocol, concomitant medications, and coexisting conditions (118–121).

Most animal studies have been performed in reductionist approaches which lack risk factors and comorbidities (122). Additional sources of variation should be considered in future studies, including the choice of anesthesia, patient’s comorbidities and comedications, and the temporal aspects of the remote conditioning algorithm (122). Caution should be exercised when assessing outcomes because patient selection and trial design may affect outcomes.

## Conclusion

Remote ischemic conditioning is protective against reperfusion injury, and further research will expand our knowledge in the field of cerebral vascular diseases. Its simplicity and non-invasive nature, as well as the flexibility of the timing of RIC stimulus, make it feasible to apply alongside neurointerventional procedures. Precise knowledge of its optimal dosage and timing of administration is yet to be found. RIC has promising but understudied potential neuroprotective influences on patients undergoing endovascular treatments who have risks of IR injury. Further validation using well-designed randomized controlled trials is necessary to document the efficacy of differing RIC protocols across a range of cerebrovascular diseases.

## Author Contributions

DK: concept, design, and development of the study; MHL: development of the study; GZ: acquisition and analysis of the data, writing of the article; GT: article writing; HTL: development of the study; RK: critical review of the article.

## Conflict of Interest Statement

The authors declare that the research was conducted in the absence of any commercial or financial relationships that could be construed as a potential conflict of interest.
